# Spermidine improves the antioxidant capacity and morphology of intestinal tissues and regulates intestinal microorganisms in Sichuan white geese

**DOI:** 10.3389/fmicb.2023.1292984

**Published:** 2024-01-16

**Authors:** Zelong Wang, Dongmei Jiang, Xin Wang, Yilong Jiang, Qian Sun, Weikang Ling, Xiaoguang An, Chengweng Ji, Shuo Li, Yuxin Qi, Bo Kang

**Affiliations:** State Key Laboratory of Swine and Poultry Breeding Industry, Farm Animal Genetic Resource Exploration and Innovation Key Laboratory of Sichuan Province, College of Animal Science and Technology, Sichuan Agricultural University, Chengdu, China

**Keywords:** spermidine, intestinal health, antioxidant capacity, microorganisms, metabolomics

## Abstract

**Introduction:**

Intestinal health is very important to the health of livestock and poultry, and is even a major determining factor in the performance of livestock and poultry production. Spermidine is a type of polyamine that is commonly found in a variety of foods, and can resist oxidative stress, promote cell proliferation and regulate intestinal flora.

**Methods:**

In this study, we explored the effects of spermidine on intestinal health under physiological states or oxidative stress conditions by irrigation with spermidine and intraperitoneal injection of 3-Nitropropionic acid (3-NPA) in Sichuan white goose.

**Results and discussion:**

Our results showed that spermidine could increase the ratio of intestinal villus to crypt and improve intestinal morphology. In addition, spermidine can also reduce malondialdehyde (MDA) accumulation caused by 3-NPA by increasing superoxide dismutase (SOD), catalase (CAT), and glutathione peroxidase (GPX) enzyme activity, thus alleviating intestinal damage. Furthermore, spermidine can regulate intestinal digestive enzyme activities and affect intestinal digestion and absorption ability. Spermidine can also promote an increase in intestinal microbial diversity and abundance and alleviate the change of microflora structure caused by 3-NPA. In conclusion, spermidine promotes the production of beneficial intestinal metabolites such as *Wikstromol*, *Alpha-bisabolol* and *AS 1–5*, thus improving the level of intestinal health. Taken together, these results indicate that spermidine can improve intestinal health by improving intestinal morphology, increasing antioxidant capacity and regulating intestinal flora structure.

## 1 Introduction

Good intestinal structure and function are the basis for various life activities of domestic animals (Zhao et al., 2023). Intestinal tissue is the largest immune organ of the body and the interface between the body and the outside world. The proliferation of antibiotics and intensive farming modes have led to more serious intestinal health problems in livestock and poultry, and intestinal tissues are vulnerable to external factors such as feed, gas and temperature, leading to oxidative stress and decreased health levels (Song et al., 2017). The intestinal health level is mainly related to the intestinal structure, antioxidant capacity and flora structure. Improving the intestinal health level of livestock and poultry has become an important way to solve the health problems of livestock and poultry in “no-resistance” breeding.

Polyamines are widely found in foods, which can be fully absorbed into the bloodstream in the gut and deposited in tissues (Eisenberg et al., 2016). The sources of polyamines in organisms can be divided into exogenous and endogenous. Exogenous polyamines come from diet and intestinal microorganisms (such as *Enterococcus faecalis* and *Campylobacter jejuni*), while endogenous polyamines come from endogenous pathways of *in vivo* synthesis and mutual transformation in cells (Yoon et al., 2023). Polyamines can improve intestinal barrier function, promote intestinal development and maintain the stability of the intestinal physiological environment (Bekebrede et al., 2020). Oral arginine (a precursor of polyamines) enhanced polyamine production by gut bacteria, suppressed systemic inflammation, improved memory, and extended the lifespan of mice. The overexpression of Ornithine decarboxylase in cells induces an increase of the α4/PP2Ac complex, which stimulates wound closure in the intestinal epithelium (Rathor et al., 2021). Song et al. (2017) showed that adding atractonide to the medium of IEC-6 cell lines could increase the contents of polyamines and Ca^2+^, thus promoting the migration and proliferation of IEC-6 cells. Cell migration and proliferation were inhibited by difluoromethyl orniorine (a polyamine synthesis inhibitor), suggesting that polyamines were involved in the regulation of IEC-6 cell migration and proliferation.

Spermidine can regulate a variety of biological processes, play a vital role in maintaining cell homeostasis, regulating immune function, promoting cell proliferation, and antioxidant functions (Madeo et al., 2018). There is a high concentration of spermidine in plant-derived food, especially in wheat germ and other feed materials (Muñoz-Esparza et al., 2021). Spermidine is a kind of drug suitable for clinical trials. It has low biotoxicity and strong effects at low and medium concentrations (Eisenberg et al., 2016). Liu et al. (2022) showed that adding 0.4 mmol/kg spermine to the diet can increase tight junction protein gene expression in the jejunum of piglets, decrease serum D-lactic acid content, and activate the Rac1/PLC-γ1 signaling pathway to protect the integrity of the intestinal barrier of piglets. Spermine supplementation during lactation can improve the intestinal villus height: crypt depth ratio and intestinal absorption area of piglets, thus promoting their growth (van Wettere et al., 2016). Spermidine can improve the antioxidant capacity of muscle cells and reduce the ratio of oxidized glutathione (GSH/GSSH) to promote cell proliferation (Ceci et al., 2022). In addition, spermidine was able to reverse the decreased activity of antioxidant enzymes in the hearts of offspring caused by hypoxia *in utero* (Chai et al., 2022). Spermidine exists in feed materials and its function is closely related to improving intestinal health, so it has great potential to be developed as a feed additive. However, the effect of spermidine on the intestinal health level of geese remains unclear.

In this study, we revealed the effects of spermidine on intestinal health under physiological states or oxidative stress conditions by irrigation with spermidine and intraperitoneal injection of 3-Nitropropionic acid (3-NPA). Our results suggest that spermidine can improve intestinal health, reduce intestinal oxidative damage, and improve intestinal flora structure. To provide basic data for the subsequent development of novel spermidine feed additives or spermidine rich diet.

## 2 Materials and methods

### 2.1 Animals ethics

All research schemes involving Sichuan white geese in this study were approved by the Animal Operation Code and Welfare Committee of Sichuan Agricultural University (No. DKY-B2020302124). The Sichuan white geese came from the Ya’an Waterfowl Breeding Farm. During the experiment, all geese were raised in a closed cage with a density of 0.5m^2^ per goose, with temperature maintained at around 25°C. They were exposed to natural light during the day. The geese had unrestricted access to feed or water.

### 2.2 Experimental design and treatment

Sichuan white geese (220 ± 5 days old) were randomly divided into four groups (n = 15). The experiment was divided into two stages. The first stage lasted for 2 days; the control group (3 mL normal saline); 3-NPA group (3 mL normal saline); spermidine (SPD) group (1 mL normal saline +2 mL 10 mg/kg SPD) and SPD + 3-NPA group (1 mL normal saline +2 mL 10 mg/kg SPD); Saline and spermidine were administered by irrigation, and the experiments were performed once in the morning and once in the evening. The second stage lasted for 5 days and included the control group (3 mL normal saline administration +2 mL normal saline intraperitoneal injection); 3-NPA group (3 mL normal saline administration +10 mg/kg 3-NPA intraperitoneal injection); SPD group (2 mL 10 mg/kg SPD + 1 mL normal saline, 2 mL normal saline by intraperitoneal injection); and 3-NPA+ SPD group (2 mL 10 mg/kg SPD + 1 mL normal saline, 2 mL 10 mg/kg 3-NPA Intraperitoneal injection).

### 2.3 Access to the intestinal tissue samples

The geese were killed by neck bleeding, all intestinal segments were quickly collected and rinsed with normal saline, and 1 cm segments were selected from the middle of each intestinal segment and held in 4% paraformaldehyde (Beyotime, Shanghai, China) for subsequent observations of intestinal morphology. The remaining intestinal samples were washed with saline, wiped with filter paper and placed in a Ziploc bag wrapped in tin foil. Isolated caecal contents were placed in EP tubes. All samples were frozen in liquid nitrogen and then placed in an ultralow temperature freezer for further testing (Wang et al., 2023).

### 2.4 Intestinal morphology analysis

Each segment was fixed with 4% paraformaldehyde (Beyotime, Shanghai, China), embedded in paraffin, sliced and stained with hematoxylin and eosin. The neutral gum is sealed and dried. Finally, the villus height and crypt depth of each intestinal segment were observed and recorded under optical microscopy (Ali et al., 2022).

### 2.5 Examination of the intestinal polyamine content

The polyamine content in intestinal tissue was determined according to the method established in the laboratory (Kang et al., 2017). An intestinal sample (0.1–0.5 mg) was placed in a glass homogenizer and ground with 5% HClO_4_ and 1, 6-hexamethylene diamine. After grinding, 12,000 g of the sample was centrifuged for 15 min. The supernatant was collected and adjusted to basic by adding 2.5 mol/L NaOH, 10 μL of benzoyl chloride was added and mixed. The sample was placed in water bath (40°C)for 1 h. The pH was adjusted to neutral by adding an appropriate amount of 6 mol/L HCl or 2.5 mol/L NaOH to the sample after the water bath. HyperSep C18 columns activated with chromatographic methanol and ultrapure water were used to extract polyamine derivatives from the sample. Finally, the samples were eluted with 15% chromatographic methanol, and the collected samples were ready for machine testing.

### 2.6 Detection of digestive enzyme activity

The intestinal tissue was removed and mashed with a moderate amount of normal saline. After centrifugation, the supernatant was taken as a sample for testing. A colorimetric method was used to determine the digestive enzyme activities in each group. The assay kits were purchased from Jiangsu Meimian Industrial Co., Ltd., Jiangsu, China. The test procedures and results are calculated in strict accordance with the instructions.

### 2.7 Malondialdehyde and antioxidant enzyme activity detection

According to the assay kit (Beyotime Biotechnology Co., Ltd., Shanghai, China), appropriate amounts of intestinal samples were taken and placed in a mortar. After liquid nitrogen was added, the corresponding prepared solution was added. After centrifugation, the supernatant was taken for testing. Superoxide dismutase (SOD), glutathione peroxidase (GPX) and catalase (CAT) enzyme activity and malondialdehyde (MDA) content were measured by colorimetry. The measurement steps and results for each index are calculated strictly as described.

### 2.8 Microbial diversity analysis of caecum

The contents of the cecum were sent to majorbio Biotechnology Co., Ltd. in Shanghai, China for 16SRNA analysis and data processing. In brief, DNA was extracted and qualified for PCR amplification. After assessing the quality and quantity of the extracted DNA, universal primers (F:ACTCCTACGGGAGGCAGCA and R:GGACTACHVGGTWTCTAATPCR) were used to amplify the V3-V4 region of the 16S rDNA, and a library was constructed. These libraries were then subjected to paired-end sequencing on the Illumina platform. Build the library and add the official splicing sequence to the outer end of the target. For computational analysis, PE reads were spliced and quality-controlled. Analysis was conducted according to the sequencing data.

### 2.9 Metabolomics analysis of caecal contents

100 μL liquid sample was added to a 1.5 mL centrifuge tube with 400 μL solution [Acetonitrile: methanol = 1:1(v:v)] containing 0.02 mg/mL internal standard (L-2-chlorophenylalanine) to extract metabolites. The samples were mixed by vortex for 30 s and low-temperature sonicated for 30 min (5°C, 40 KHz). The samples were placed at −20°C for 30 min to precipitate the proteins. Then the samples were centrifuged for 15 min (4°C, 13,000 g). The supernatant was removed and blown dry under nitrogen. The sample was then re-solubilized with 100 μL solution (acetonitrile: water = 1:1) and extracted by low-temperature ultrasonication for 5 min (5°C, 40 KHz), followed by centrifugation at 13,000 g and 4°C for 10 min. The supernatant was transferred to sample vials for LC-MS/MS analysis. The LC-MS/MS analysis of sample was conducted on a Thermo UHPLC-Q Exactive HF-Xsystem equipped with an ACQUITY HSS T3 column (100 mm × 2.1 mm i.d., 1.8 μm; Waters, United States) at Majorbio Bio-Pharm Technology Co. Ltd. (Shanghai, China). The mobile phases consisted of 0.1% formic acid in water: acetonitrile (95:5, v/v; solvent A) and 0.1% formic acid in acetonitrile: isopropanol: water (47.5:47.5, v/v; solvent B). The flow rate was 0.40 mL/min and the column temperature was 40°C. After the mass spectrometry detection is completed, the raw data of LC/MS is preprocessed by Progenesis QI (Waters Corporation, Milford, United States) software, and a three-dimensional data matrix in CSV format is exported. Perform variance analysis on the matrix file after data preprocessing. Differential metabolites among two groups were summarized, and mapped into their biochemical pathways through metabolic enrichment and pathway analysis based on database search (KEGG, http://www.Genome.jp/kegg/).

### 2.10 Statistical analysis

The data were collated and analyzed by EXCEL, and the results are expressed as mean ± SD. The ANOVE program was used for significance analysis, *p* < 0.05 indicated a significant difference, and GraphPad Prism was used for mapping. The experimental data consist of three replicas.

## 3 Results

### 3.1 Intestinal morphological change

**Figure 1:**
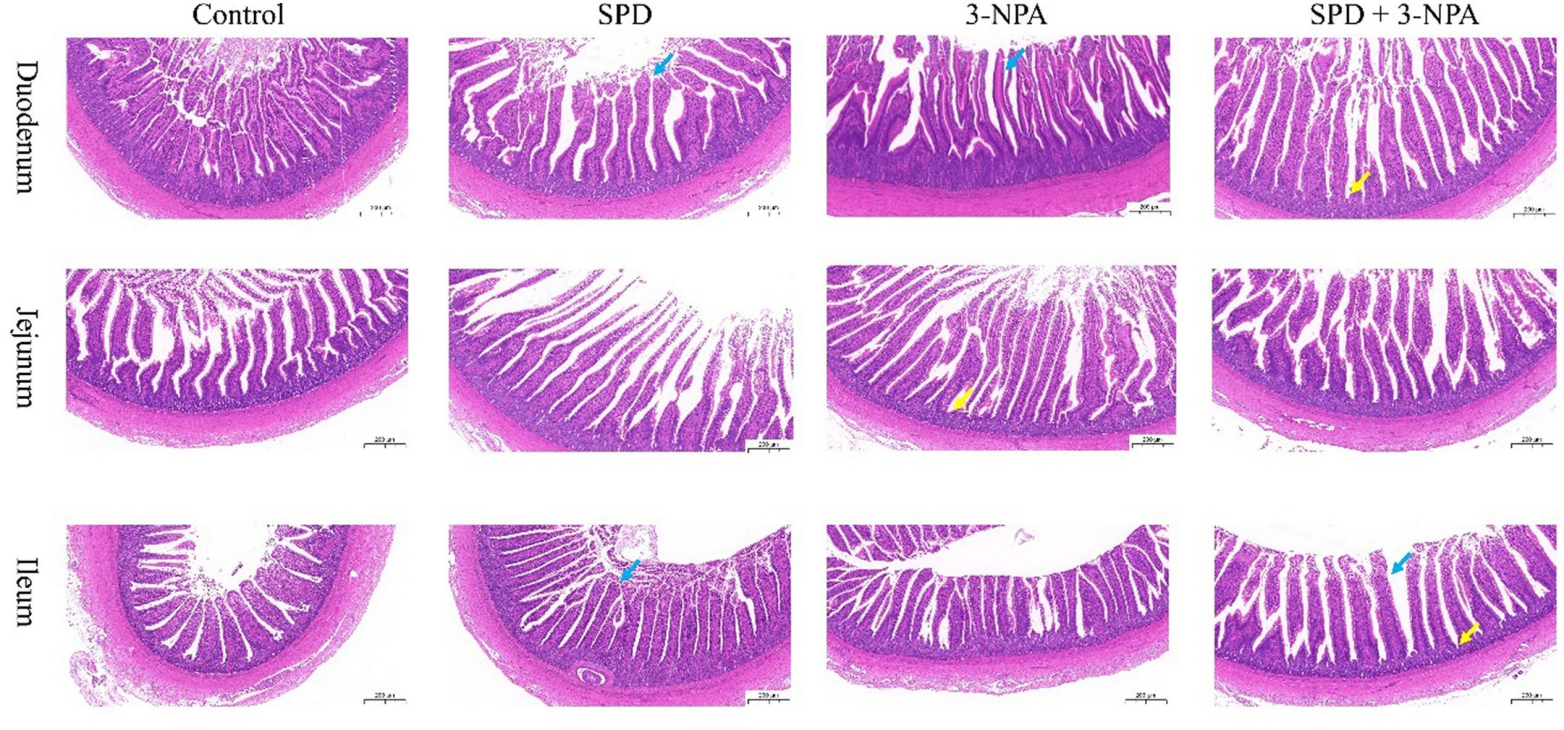
Effects of spermidine on intestinal morphology. Pictures were observed at 200× magnification. The blue arrow indicates intestinal villi and the yellow arrow indicates crypts.

The villus height of the duodenum, jejunum and ileum in the SPD group was significantly higher than that in the control group, and the ratio of villus to crypt was also significantly increased (*p* < 0.05). The duodenal villus height in the 3-NPA group was significantly increased, and the jejunum and ileum villus height/crypt depth were significantly higher than that in the control group (*p* < 0.05). Compared with the 3-NPA group, the villus height and correct ratio of the duodenum and ileum in the SPD + 3-NPA group were significantly increased (*p* < 0.05; Figure 1; Table 1).

**Table 1:** Effects of spermidine on intestinal morphological parameters.

Item	Control	SPD	3-NPA	SPD + 3-NPA	*p*-value
**Duodenum**					
VH, μm	668.05 ± 150.85^c^	1079.22 ± 80.11^a^	843.56 ± 46.05^b^	1142.69 ± 71.75^a^	<0.0001
CD, μm	146.75 ± 14.18^a^	123.23 ± 9.26^b^	141.08 ± 24.06^a^	112.41 ± 27.69^b^	0.0571
VH: CD	4.63 ± 1.36^b^	8.81 ± 1.53^a^	6.12 ± 1.03^b^	10.60 ± 2.32^a^	<0.0001
**Jejunum**					
VH, μm	865.67 ± 139.64^b^	1624.78 ± 99.73^a^	954.95 ± 165.75^a^	879.78 ± 175.85^a^	<0.0001
CD, μm	112.17 ± 15.66^a^	67.16 ± 7.60^c^	88.90 ± 7.70^b^	87.92 ± 12.75^b^	0.0002
VH:CD	7.93 ± 1.98^c^	24.44 ± 3.03^a^	10.72 ± 1.52^b^	10.35 ± 3.54^b^	<0.0001
**Ileum**					
VH, μm	752.14 ± 46.87^c^	1009.93 ± 53.57^b^	798.50 ± 71.70^c^	1143.16 ± 34.66^a^	<0.0001
CD, μm	106.2 ± 14.84^ab^	112.55 ± 9.22^a^	92.77 ± 10.51^bc^	87.50 ± 6.43^c^	0.0066
VH: CD	7.16 ± 0.81^c^	9.00 ± 0.61^b^	8.76 ± 1.77^b^	13.10 ± 0.73^a^	<0.0001

VH, Villus height; CD, Crypt depth. The data are presented as the mean ± SD. ^a,b^Means in the same column with different superscripts are significantly different by Student’s test (*p* < 0.05).

### 3.2 The intestinal polyamine content changes

**Figure 2:**
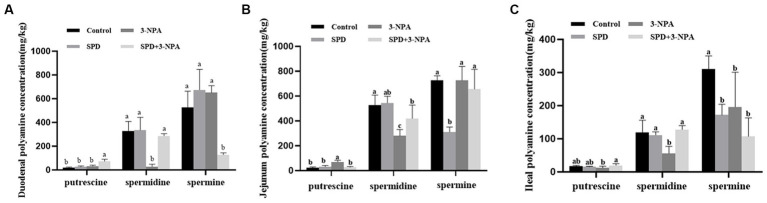
Effect of spermidine on polyamine content in the intestinal tract **(A)** duodenum; **(B)** jejunum; **(C)** ileum. Different superscript letters were significantly different by Student’s test (*p* < 0.05).

The contents of putrescine and spermidine in the duodenum, jejunum and ileum of the SPD group were not significantly different from those of the control group (*p* > 0.05). However, the contents of spermidine in the jejunum and ileum of the SPD group were significantly lower than those of the control group (*p* < 0.05). The contents of spermine in the jejunum and ileum of the SPD group were significantly lower than those of the control group (*p* < 0.05). The content of spermidine in the duodenum, jejunum and ileum in the 3-NPA group was significantly lower than that in the control group (*p* < 0.05). Compared with the 3-NPA group, spermidine content in the small intestine and putrescine content in the duodenum and ileum in the SPD + 3-NPA group were significantly increased, but spermine content in the duodenum were significantly decreased (*p* < 0.05; Figures 2A–C).

### 3.3 Digestive enzyme activity changes

The duodenal amylase activity of the SPD group was significantly higher than that of the control group (*p* < 0.05), and the duodenal and jejunal lipase activity was significantly lower than that of the control group (*p* < 0.05). The activity of amylase in the jejunum of the 3-NPA group was significantly higher than that of the control group (*p* < 0.05), but there was no significant difference in the activity of lipase in duodenum, jejunum and ileum between the 3-NPA group and the control group (*p* > 0.05). Compared with the 3-NPA group, the amylase activities in the duodenum and ileum in the SPD + 3-NPA group were significantly decreased (*p* < 0.05), and the trypsin activities in the jejunum and ileum were significantly decreased (*p* < 0.05; Table 2).

**Table 2:** Effects of spermidine on intestinal digestive enzyme activity.

Item	Control	SPD	3-NPA	SPD + 3-NPA	*P-*value
**Duodenum**					
Trypsin	6.18 ± 0.59^a^	5.77 ± 0.52^a^	5.86 ± 0.07^a^	6.15 ± 0.74^a^	0.6389
Amylase	14.76 ± 1.17^b^	17.71 ± 1.38^a^	15.01 ± 0.68^b^	12.29 ± 1.16^c^	0.0002
Lipase	26.94 ± 0.53^a^	20.02 ± 2.12^b^	26.57 ± 1.73^a^	25.84 ± 4.53^a^	0.2675
**Jejunum**					
Trypsin	6.27 ± 0.42^a^	5.89 ± 0.23^a^	6.37 ± 0.58^a^	5.23 ± 0.21^b^	0.1241
Amylase	15.80 ± 0.67^b^	16.02 ± 0.89^b^	20.31 ± 1.23^a^	14.34 ± 0.47^c^	<0.0001
Lipase	28.54 ± 1.25^a^	21.81 ± 2.17^b^	26.50 ± 0.54^a^	26.04 ± 2.27^a^	0.0010
**Ileum**					
Trypsin	5.64 ± 0.27^b^	6.21 ± 0.28^a^	5.03 ± 0.21^c^	4.59 ± 0.25^d^	<0.0001
Amylase	14.53 ± 1.17^ab^	15.68 ± 1.31^a^	13.03 ± 1.33^b^	14.16 ± 0.70^ab^	0.0476
Lipase	19.35 ± 0.84^a^	20.74 ± 1.24^a^	18.85 ± 2.79^a^	18.09 ± 1.14^a^	0.2071

The data are presented as the mean ± SD. ^a–d^Means in the same column with different superscripts are significantly different by Student’s test (*P* < 0.05).

### 3.4 Intestinal MDA and antioxidant enzyme activity changes

The activities of SOD in the jejunum and ileum, and GPX in the duodenum, jejunum and ileum in the SPD group were significantly higher than those in the control group (*p* < 0.05). The SOD activity of the duodenum in the 3-NPA group was significantly lower than that in the control group (*p* < 0.05), and the SOD activity of the jejunum and ileum and the CAT activity of the duodenum, jejunum and ileum in the 3-NPA group were significantly higher than that in control group (*p* < 0.05). Compared with the 3-NPA group, SOD activity in the duodenum, jejunum and ileum in the SPD + 3-NPA group was significantly increased (*p* < 0.05). In addition, the MDA content in the duodenum, jejunum and ileum in the SPD group was significantly lower than that in the control group (*p* < 0.05). MDA levels in the duodenum, jejunum and ileum in the 3-NPA group were significantly higher than those in the control group (*p* < 0.05). Compared with that in the 3-NPA group, the MDA content in duodenum, jejunum and ileum in the SPD + 3-NPA group was significantly decreased (*p* < 0.05; Table 3).

**Table 3:** Effects of spermidine on antioxidant enzyme activity and MDA.

Item	Control	SPD	3-NPA	SPD + 3-NPA	*P*-value
**Duodenum**					
SOD, U/mg	25.02 ± 2.10^a^	22.73 ± 2.73^ab^	20.38 ± 2.87^c^	22.20 ± 2.52^ab^	0.0678
GPX, U/mg	59.45 ± 4.86^b^	90.74 ± 6.90^a^	54.13 ± 6.68^b^	76.29 ± 12.5^0a^	0.0010
CAT, U/mg	8.23 ± 0.73^b^	8.13 ± 0.68^b^	11.84 ± 0.22^a^	10.23 ± 0.73^a^	0.0002
MDA, μmol/mg	9.33 ± 0.47^c^	7.09 ± 0.20^d^	12.79 ± 0.24^a^	11.39 ± 0.55^b^	<0.0001
**Jejunum**					
SOD, U/mg	11.53 ± 0.52^b^	13.15 ± 1.11^a^	12.93 ± 1.00^a^	13.54 ± 0.30^a^	0.0128
GPX, U/mg	39.22 ± 2.15^c^	74.72 ± 5.38^a^	45.60 ± 9.47^bc^	60.92 ± 4.03^ab^	0.0134
CAT, U/mg	5.67 ± 0.59^b^	5.32 ± 0.74^b^	7.69 ± 0.54^a^	8.53 ± 0.53^a^	0.0005
MDA μmol/mg	17.63 ± 1.77^b^	7.13 ± 1.68^d^	20.52 ± 3.01^a^	11.08 ± 1.20^c^	<0.0001
**Ileum**					
SOD, U/mg	20.60 ± 1.50^b^	25.39 ± 0.79^a^	23.37 ± 1.82^a^	23.42 ± 2.20^a^	0.0226
GPX, U/mg	57.23 ± 8.06^b^	152.74 ± 20.25^a^	120.33 ± 26.46^b^	78.66 ± 9.33^a^	0.0007
CAT, U/mg	6.08 ± 0.12^b^	7.57 ± 0.20^a^	7.47 ± 0.62^a^	6.47 ± 0.63^b^	0.0004
MDA, μmol/mg	9.30 ± 1.07^b^	5.42 ± 0.19^c^	12.99 ± 0.48^a^	8.99 ± 0.65^b^	<0.0001

SOD, Superoxide dismutase; GPX, Glutathione peroxidase; CAT, Catalase; MDA, Malondialdehyde. The data are presented as the mean ± SD. ^a–d^Means in the same column with different superscripts are significantly different by Student’s test (*P < 0.05*).

### 3.5 Microbe distribution in the caecum of Sichuan white geese

**Figure 3:**
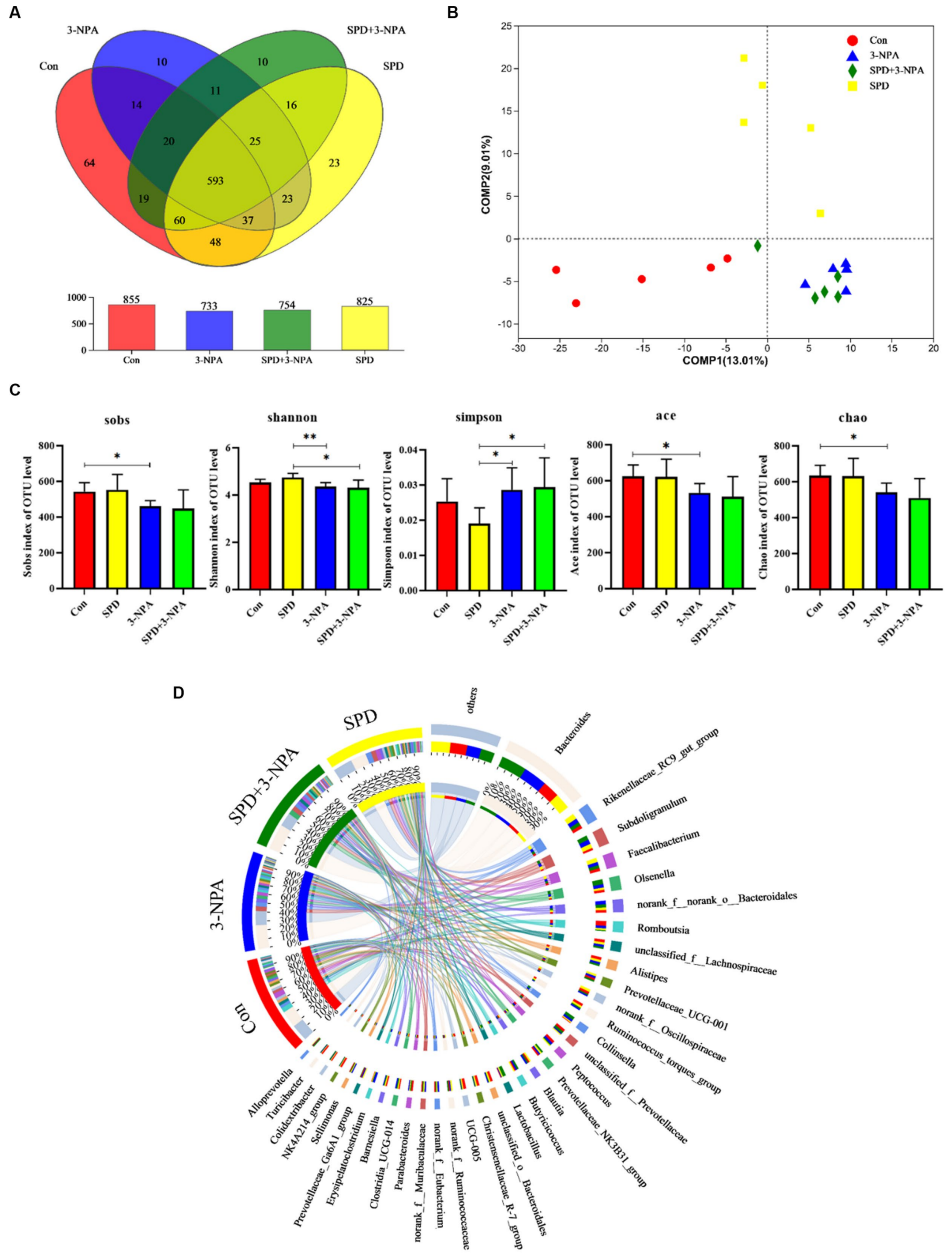
Effect of spermidine on intestinal flora structure. **(A)** Venn diagram; **(B)** Principal coordinate analysis based on OTU level; **(C)** The α-diversity of the microbial community, bar with the asterisk (*) level suggests the degree of significant difference, and the values are indicated as the means ± SD (^*^ *p* < 0.05, ^**^p < 0.01). **(D)** The discriminant analysis of LEfSe multilevel species differences from the phylum to genus level.

The caecal contents of five geese from each group were randomly selected for sequencing to assess microbial diversity. After quality control of sequencing data, an average of 43,426 optimized sequences were obtained for each sample, with a length of 416 bp. Based on 97% species similarity, the operational taxonomic units (OTU) picking with was compiled with Qiime using default parameters. Taxonomic classification was performed based on the OTU database. The sequencing sequences were clustered through the Uparse software platform and 97% of similar sequences were divided into an OUT (Operational Taxonomic Units) and a total of 3,167 OTUs were obtained. Among them, 593 were core OTUs, 64 OTUs were unique to the control group, 23 OTUs were unique to the SPD group, 10 OTUs were unique to the 3-NPA group, and 10 OTUs were unique to the SPD + 3-NPA group (Figure 3A). The PCoA map reflected the difference (OTU level) in caecal microflora in each treatment groups, While the 3-NPA group and the SPD + 3-NPA group had a large overlap, between the three treatment groups and the control group showed clear separation from them. These results indicated that spermidine and 3-NPA could affect the structure of the intestinal flora, but spermidine had a limited effect on the structure of the intestinal flora induced by 3-NPA. 3-NPA treatment significantly decreased the Sobs index, Ace index and Chao index compared with control group (*p* < 0.05). The results showed that 3-NPA treatment significantly reduced the diversity of caecal microflora. The 3-NPA group and the SPD + 3-NPA group there was no significant difference of microbial diversity, and spermidine cannot affect the microbial diversity decline caused by 3-NPA. Compared with the control group, the 3-NPA group showed better aggregation, which may be related to the effect of 3-NPA on the colonization of some microflora in the caecum and the reduction in microflora diversity (Figure 3B). 3-NPA treatment significantly decreased the Sobs, Ace and Chao index compared with control group (*p* < 0.05). The results showed that 3-NPA treatment significantly reduced the diversity of caecal microflora. Compared with the 3-NPA group and the SPD + 3-NPA group, the Shannon index in the SPD group was significantly increased, and the Simpson index was significantly decreased (*p* < 0.05; Figure 3C). These results indicated that spermidine and 3-NPA could affect the structure of the intestinal flora, but spermidine had a limited effect on the structure of the intestinal flora induced by 3-NPA. A Circos map was used to display the dominant flora and the proportion of individual flora in each treatment groups and to visualize the influence of different treatments on the proportion of intestinal flora at the genus level (Figure 3D).

**Figure 4:**
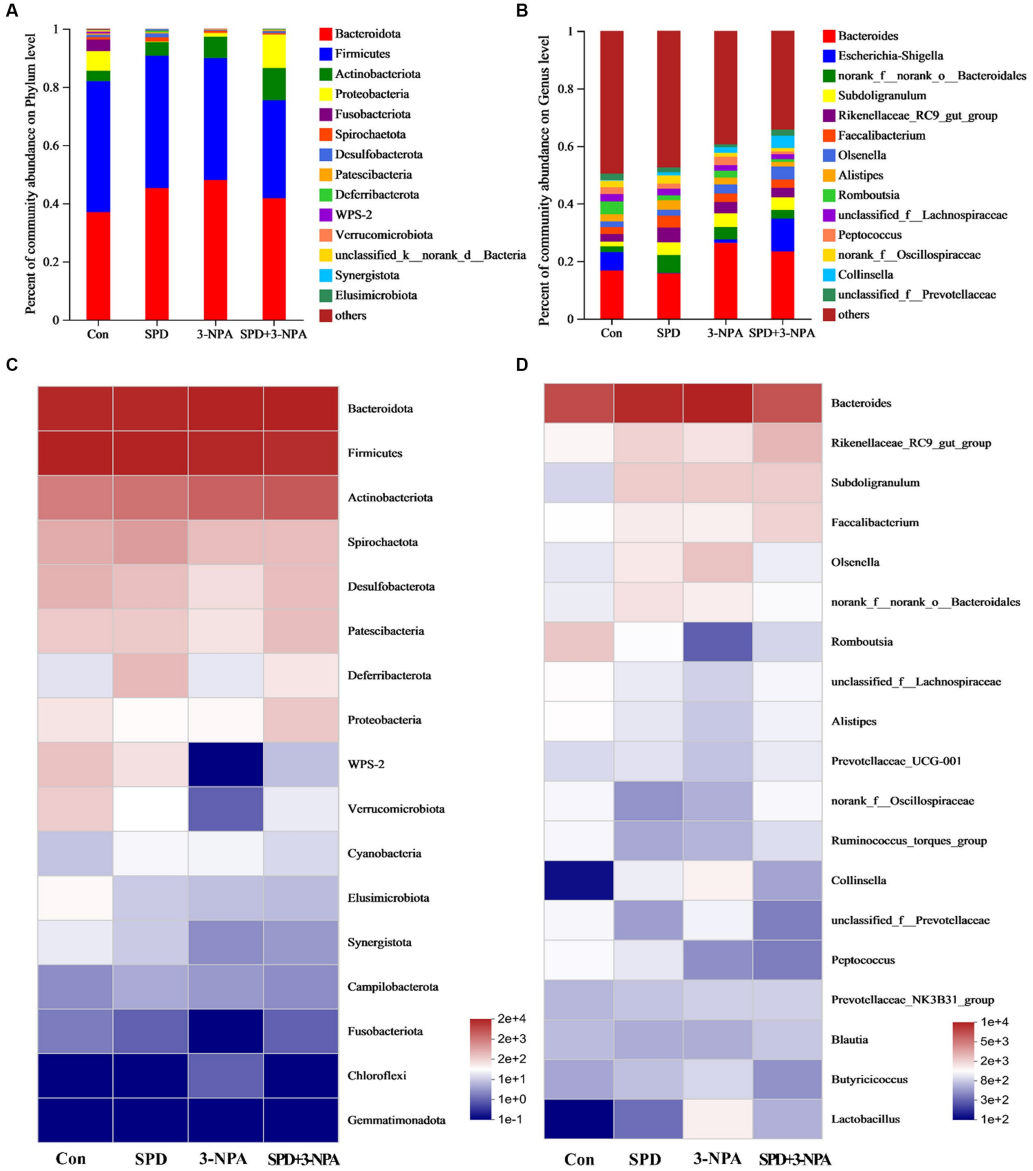
Effects of spermidine on intestinal flora abundance. **(A,B)** Barplot analysis of microbial community compositions at the phylum and genus levels. **(C,D)** Heatmap analysis of microbial community compositions at the phylum levels and genus levels.

*Firmicutes* and *Bacteroidetes* accounted for 90% of the total microflora in the caecum of geese. At the phylum or genus level, 3-NPA changed the structure of intestinal flora and decreased the diversity of intestinal flora. The intestinal flora structure of SPD group was similar to that of the control group (Figure 4A). At the genus level, the microorganisms that dominated the top five in the control group were *Bacteroides* (18.25%), *Romboutsia* (5.18%), *Rikenellaceae_RC9_gut_group* (3.20%), *unclassified_f__Lachnospiraceae* (2.95) and *Alistipes* (2.9%). In the SPD group were *Bacteroides* (16.87%), *Rikenellaceae_RC9_gut_group (6.06%)*, *Subdoligranulum* (4.86%), *Faecalibacterium* (4.58%) and *norank_f__norank_o__Bacteroidales* (2.74%); in the 3-NPA group were *Bacteroides* (25.67%), *Subdoligranulum* (4.91%), *Rikenellaceae_RC9_gut_group* (4.57%) and *norank_f__norank_o__Bacteroidales* (3.91%) and *Olsenella* (3.63%); in the SPD + 3-NPA group were *Bacteroides* (27.82%), *Olsenella* (5.34%), *Subdoligranulum* (4.92%), *Rikenellaceae_RC9_gut_group* (3.88%) and *norank_f__norank_o__Bacteroidales* (3.47%; Figure 4B).

To intuitively see the difference in the bacterial community among intestinal samples of Sichuan white geese, we conducted species heatmap clustering analysis. At the phylum level, *Bacteroidetes*, *Firmicutes* and *Actinobacteria* were abundant in the intestinal flora. *Gemmatimonadota* abundance is low. Treatment with 3-NPA reduced the abundance of *WPS-2* and *Verrucomicrobiota* in the gut. Spermidine slightly alleviated 3-NPA induced intestinal flora changes. The changes in flora abundance at the genus level and phylum level were different in the intestinal flora of Sichuan white geese. Spermidine or 3-NPA increased the abundance of *Rikenellaceae_RC9_gut_group*, *Subdoligranulum* and *Faecalibacterium*. Interestingly, spermidine or 3-NPA did not improve the bacterial abundance of *Collinsella* and *Lactobacillus*, but spermidine combined with 3-NPA increased the bacterial abundance (Figures 4C,D).

**Figure 5:**
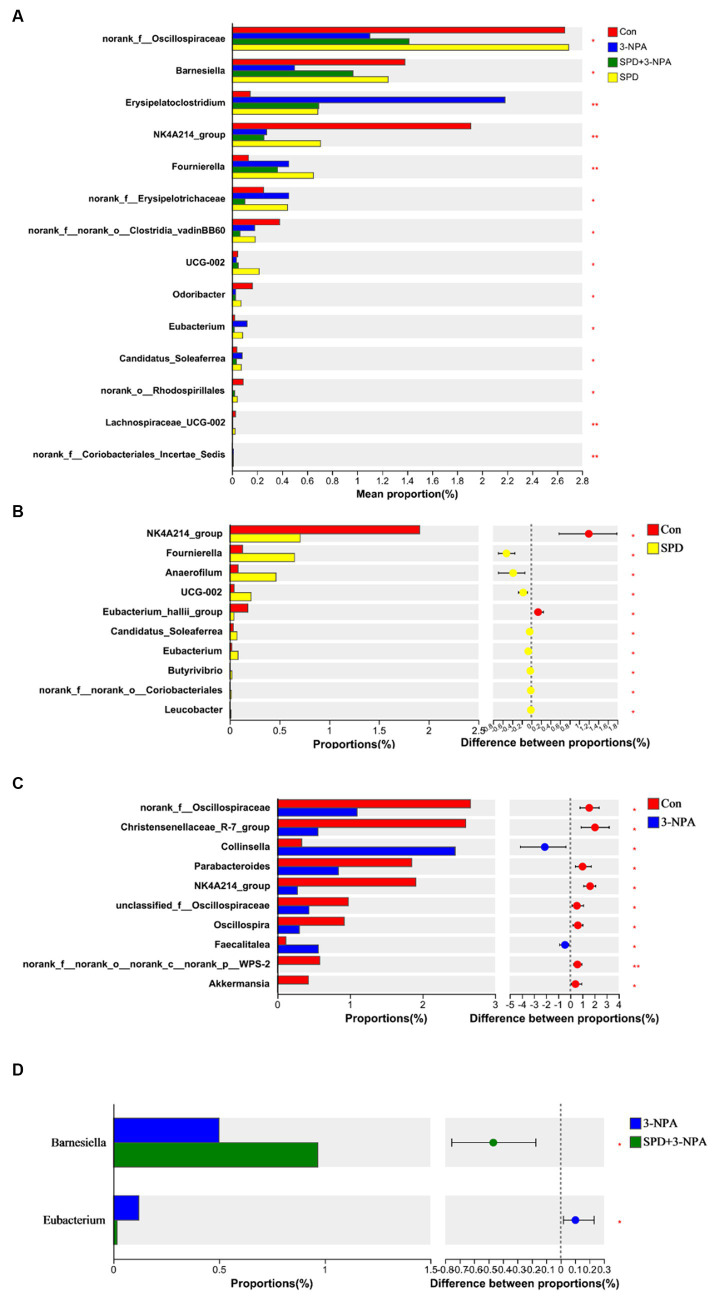
Differences in intestinal flora among different groups at the genus level. **(A)** Significance test of differences between treatments at the genus level. **(B)** Control group and SPD group. **(C)** Control group and 3-NPA group. **(D)** SPD group and SPD + 3-NPA group. Bar with the asterisk (^*^p < 0.05, ^**^p < 0.01).

Based on the community abundance data in the sample, we used statistical methods to detect species with abundance differences in each group of flora, and assessed the significance of the differences. *norank_f__Oscillospiraceae*, *Barnesiella*, *Erysipelatoclostridium*, *NK4A214*, *Fournierella* and *Clostridia_vadinBB60* showed significant differences among the treatment groups (*p* < 0.05). To further understand the effects of each treatment on the intestinal flora, two groups were selected for comparison. The abundances of *Fournierella* and *Anaerofilum* in the SPD group were significantly higher than those in the control group, while the abundances of *Eubacterium_hallii_group* and *NK4A214_group* were significantly lower than those in the control group (*p < 0.05*; Figures 5A,B). Compared with the control group, the abundance of *norank_f__Oscillospiraceae*, *Christensenellaceae_R-7_group*, *Parabacteroides* and *NK4A214* decreased significantly in the 3-NPA group *(p < 0.05)*. The abundance of *Collinsella* and *Faecalitalea* increased significantly *(p < 0.05)*. Compared with the 3-NPA group, the abundance of *Barnesiella* in the 3-NPA + SPD group significantly increased, while the abundance of *Eubacterium* significantly decreased (*p < 0.05*; Figures 5C,D).

### 3.6 Effects of spermidine on metabolites of intestinal flora

**Figure 6:**
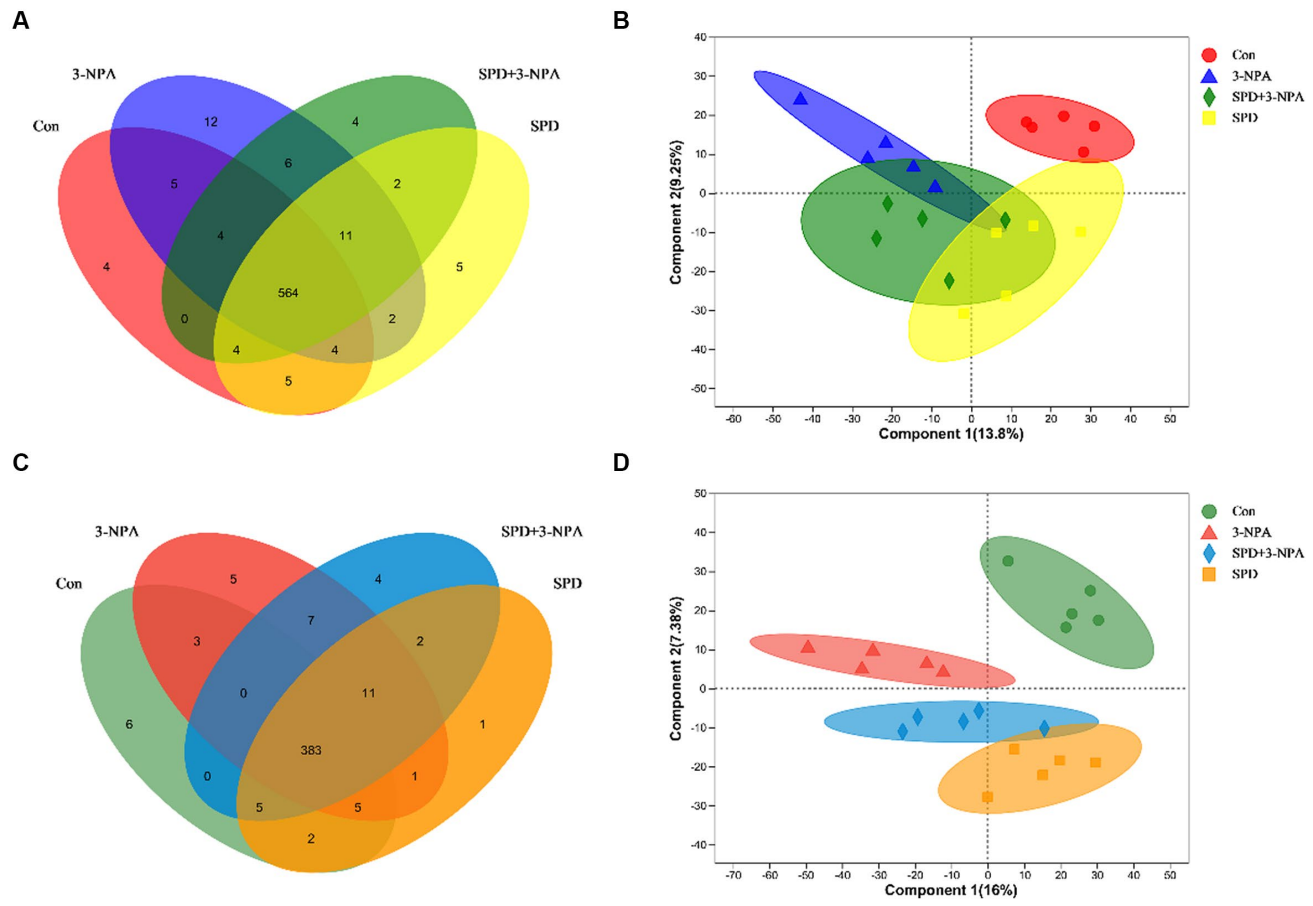
Effects of spermidine on intestinal metabolites. **(A)** Venn diagram of cationic metabolites. **(B)** PLS-DA analysis of cationic metabolites. **(C)** Venn diagram of anion metabolites. **(D)** PLS-DA analysis of anionic metabolites.

**Figure 7:**
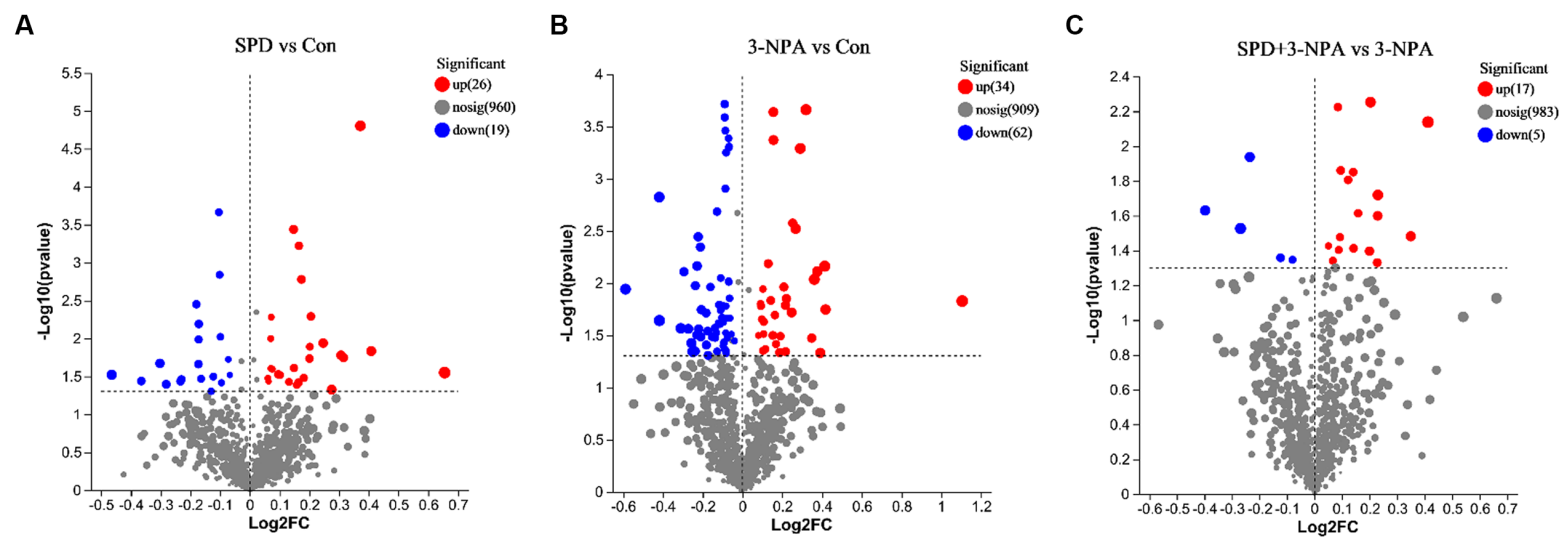
Volcanic maps of different metabolites of each group. **(A)** Control group and SPD group. **(B)** Control group and 3-NPA group. **(C)** SPD + 3-NPA group and 3-NPA group.

A total of 9,785 positive ion peaks and 592 metabolites were detected by metabolome sequencing. There were 9,694 negative ion peaks and 413 metabolites (Table 4). The Venn diagram provides a clear understanding of the metabolites that exist between treatment groups and those that are unique to each group (Figures 6A,C). PLS-DA reflected the difference in metabolites in each treatment group. In the cationic mode, there was a partial overlap between the SPD group and the control group. There was overlap between the 3-NPA group and the SPD + 3-NPA group. In anionic mode, the SPD group and control group were separated. There was a small overlap between the 3-NPA group and the SPD + 3-NPA group (Figures 6B,D). To further explore the effects of spermidine, 3-NPA and oxidative stress on intestinal metabolites, differential metabolites were screened by limiting the variable Important in the projection, fold change and *p* value. When VIP > 1 and p value <0.05, 208 different metabolites were screened. There were 45 different metabolites in the SPD group and the control group, of which 26 metabolites were significantly increased and 19 metabolites were significantly decreased. There were 96 different metabolites in the 3-NPA group and the control group, among which 34 metabolites were significantly increased and 62 were significantly decreased. There were 22 different metabolites in the combined treatment group and 3-NPA group, among which 17 metabolites were significantly increased and 5 metabolites were significantly decreased (Table 5; Figures 7A–C).

**Table 4:** Statistical table of total ions and identification of intestinal metabolites in Sichuan white goose.

Lon mode	All peaks	Identified metabolites	Metabolites in Library	Metabolites in KEGG
Pos	9,785	592	463	206
Neg	9,694	413	378	114

**Table 5:** Statistics of different metabolites in each treatment group.

Group name	All difference	Decreased	Increased
Control vs. SPD	45	19	26
Control vs. 3-NPA	96	62	34
SPD + 3-NPA vs. 3-NPA	22	5	17

**Figure 8:**
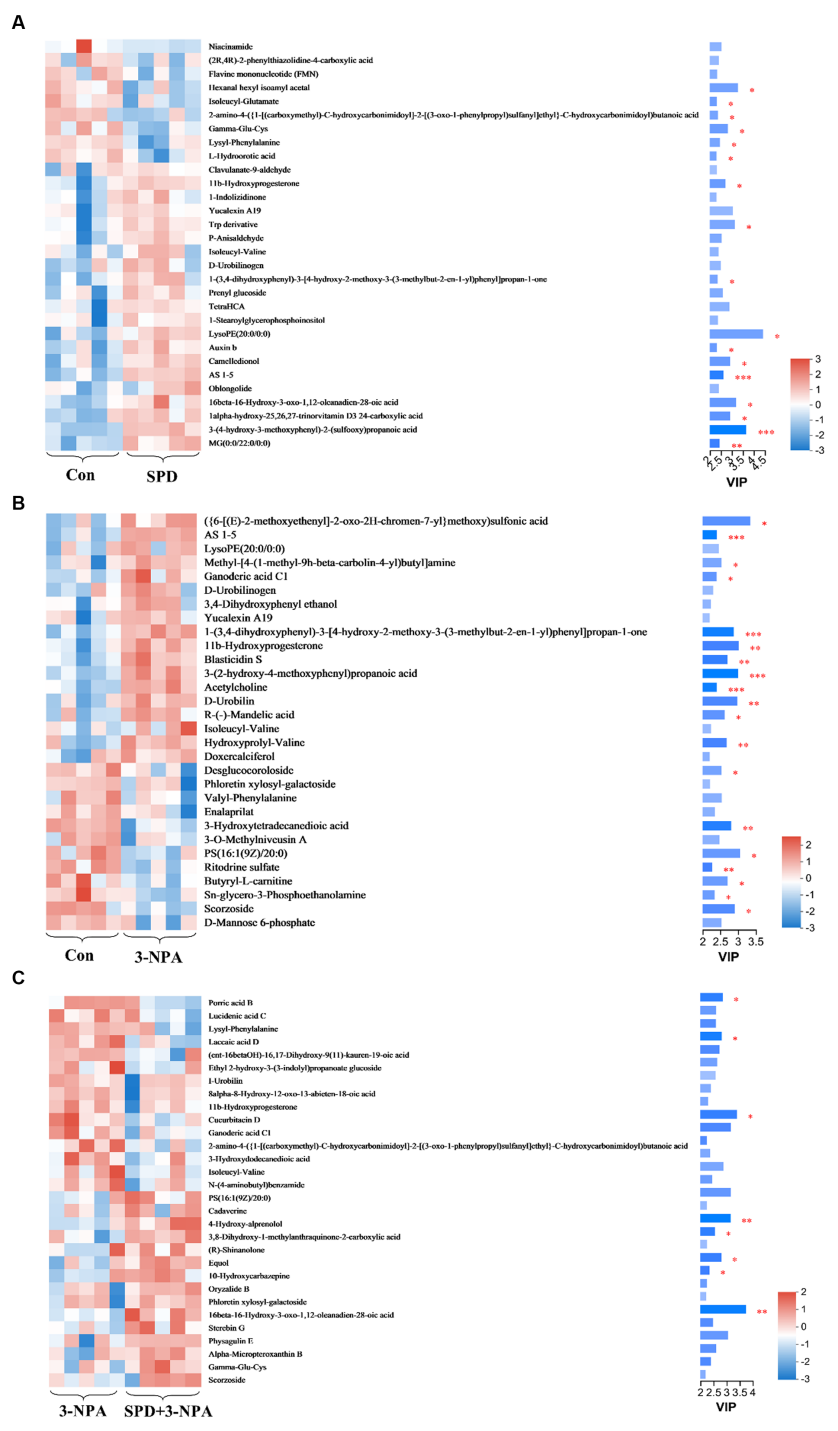
Heatmap of different metabolites in each group. **(A)** Control group and SPD group. **(B)** Control group and 3-NPA group. **(C)** SPD group and SPD + 3-NPA group. Bar with the asterisk (^*^p < 0.05, ^**^p < 0.01, ^***^p < 0.001).

The top 30 relative abundances of intestinal metabolites in the 3-NPA group, the SPD group, the SPD + 3-NPA group and the control group were selected to draw a cluster heatmap and VIP bar chart to further explore the changes in intestinal metabolites. The contents of *Flavine mononucleotide*, *PE (15:0/16:0)*, *Laccaic acid D* and other metabolites in the SPD group were significantly lower than those in the control group (*p < 0.05*). The contents of *Wikstromol*, *N-adaverine*, *Alpha-bisabolol* and *AS 1–5* were significantly increased (*p < 0.05*; Figure 8A). The contents of *Isodesmosine*, *Blasticidin S*, *Bufotenin*, *D-Urobilin*, *Aucubin*, *N-butylscopolamine* and *Lysyl-phenylalanine* in the 3-NPA group were significantly higher than those in the control group; The levels of D-mannose 6-phosphate and Butyryl-L-carnitine were significantly reduced (*p < 0.05*; Figure 8B). Compared with the 3-NPA group, the content of Ferulic acid 4-sulfate in the SPD + 3-NPA group was significantly decreased *(p < 0.05)*. Phloretin xylosyl-galactoside, Equol, Sphingosine, and 17-phenyl trinor prostaglandin F2alpha serinol amide content was significantly increased (*p < 0.05*; Figure 8C).

**Figure 9:**
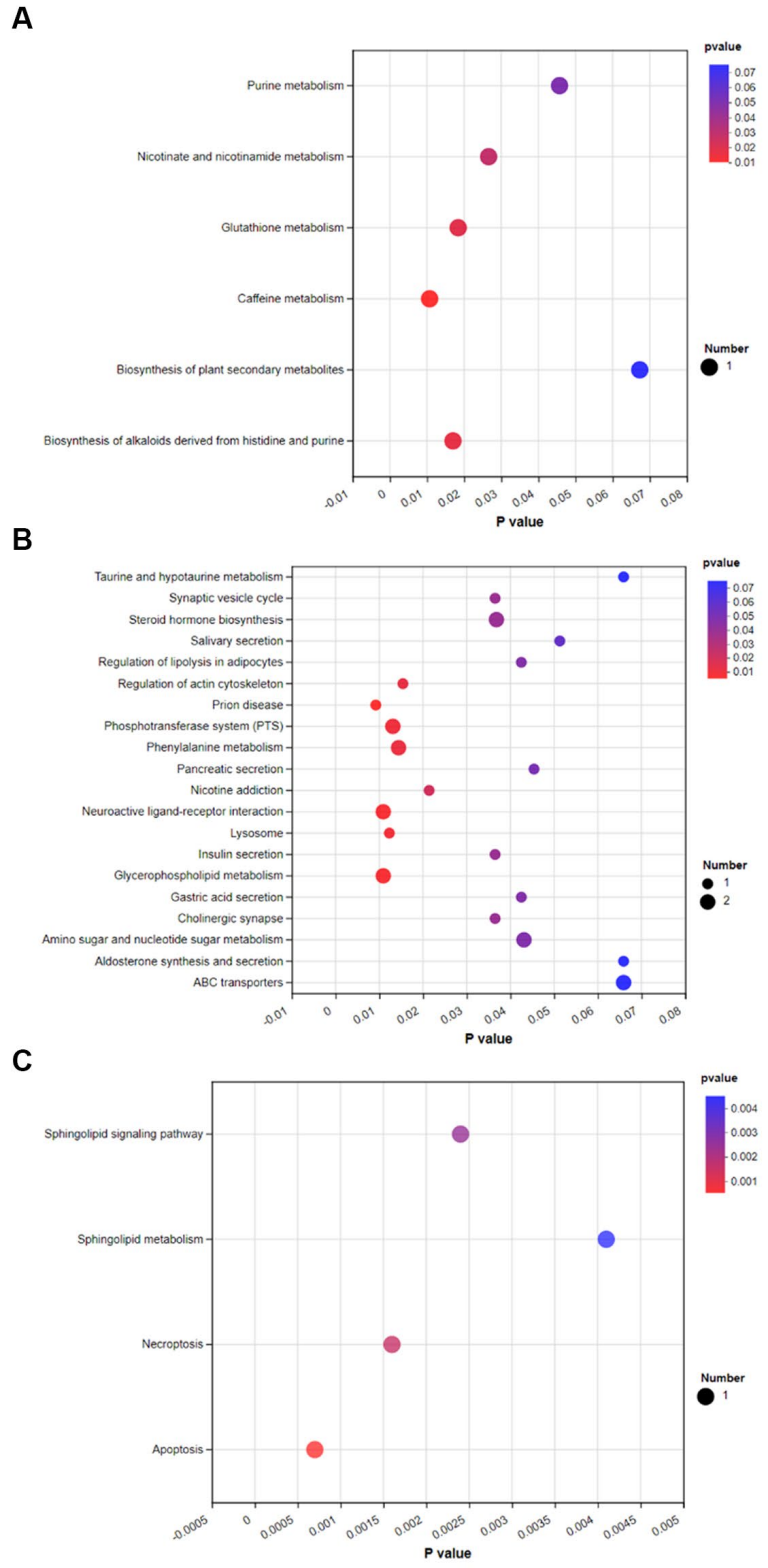
Bubble map of KEGG enrichment of different metabolites in each group. **(A)** Control group and SPD group. **(B)** Control group and 3-NPA group. **(C)** SPD group and SPD + 3-NPA group.

To investigate the effects of spermidine and 3-NPA on metabolic pathways, KEGG enrichment analysis was performed. As shown in Figure 9A, spermidine treatment had significant effects *(p < 0.05)* on caffeine metabolism, Glutathione metabolism, Biosynthesis of alkaloids derived from histidine and purine, and Nicotinate and nicotinamide metabolism and Purine metabolism. 3-NPA enriched Prion disease, Neuroactive ligand–receptor interaction, Phosphotransferase system, and Regulation of actin. The pathways of cytoskeleton and Phenylalanine metabolism was significantly affected (*p < 0.05*; Figure 9B). Compared with the 3-NPA group, the SPD + 3-NPA group significantly affected the signaling pathway, Sphingolipid metabolism, Necroptosis and Apoptosis pathways (*p < 0.05*; Figure 9C).

## 4 Discussion

The gut is the main place for animals to absorb nutrients from the outside world, and its basic structure includes crypts and villi. Intestinal villi expand the intestinal surface area and enhance absorption (Kai, 2021). Wei et al. (2022) found that 8 μmol/L spermidine can promote the proliferation of IPEC-J2 cells. This study found that spermidine could improve the villus height and villus-crypt ratio in the gut of geese. The ratio can also reflect intestinal absorption capacity. It is speculated that spermidine can improve the height of intestinal villi by promoting cell proliferation, thus enhancing digestion and absorption. 3-NPA also increased the villus height of the duodenum and the villus height: crypt depth ratio of the jejunum and ileum. However, our subsequent experiments found that 3-NPA treatment can induce an increase in intestinal MDA content, resulting in intestinal oxidative damage. 3-NPA can significantly increase the expression of inflammatory factors and related proteins in glial cells, resulting in inflammatory response (Jang et al., 2023). It is speculated that 3-NPA can induce intestinal oxidative stress or inflammation, resulting in intestinal tissue enlargement, resulting in an increase in intestinal villus-crypt ratio. Compared with the 3-NPA group, the villus height of the duodenum and ileum was significantly increased in the SPD + 3-NPA group. We found that the contents of spermidine and putrescine in the duodenum and ileum were significantly increased in the group treated with spermidine combined with 3-NPA. Putrescine plays an important role in protecting intestinal integrity and promoting epithelial cell maturation (Liu et al., 2019). The contents of spermidine and putrescine increased in the group treated with spermidine combined with 3-NPA. Interestingly, the contents of putrescine and spermidine increased in the group treated with spermidine combined with 3-NPA, while the contents of spermidine in the duodenum and ileum decreased. There was no significant change in the contents of putrescine and spermidine in each intestinal segment of the SPD group, but the contents of spermidine in the jejunum and ileum decreased. It is speculated that exogenous spermidine does not affect the metabolism of putrescine and spermidine in physiological state. Pegg and Erwin (1985) found that after intraperitoneal injection of spermidine, the activity of spermidine/Spermine N1-acetyltransferase (SSAT) in the liver, kidney and lung tissue of mice increased. SSAT can catalyze the transfer of acetyl groups to spermidine or spermine amidopropyl terminals to produce N1-acetylated spermidine or spermine (Kurihara, 2022). It was speculated that feeding spermidine could improve the activity of SSAT in the gut of geese and lead to the conversion of spermidine to N1-acetylated spermidine deposition. In conclusion, feeding spermidine can improve intestinal morphology and increase villus-cyrpt ratio.

Digestive enzyme activity can affect intestinal digestion and intestinal function. Poultry digestive enzymes are mainly secreted by the pancreas and distributed mainly in the duodenum and jejunum (Gu et al., 2021). Amylase helps the gut digest gluten in food (Smith et al., 2015). Our study found that spermidine can increase the activity of amylase in the duodenum, suggesting that exogenous supplementation with spermidine is conducive to gluten decomposition. Lipase can participate in the digestion of fats and lipids and regulate gallbladder function (Patel et al., 2019). Reducing lipase activity an effective means to treat obesity. Lipase inhibitors change the lipid metabolism of the body by combining with active lipase in the intestine, thus reducing the digestion, absorption and accumulation of lipids in food (Liu et al., 2020). Zhang et al. (2020) showed that inhibition of lipase could improve the activity of immune enzymes, antioxidant capacity and the expression of anti-inflammatory factors, and improve the adverse effects caused by a high-fat diet. Our study found that feeding spermidine reduced duodenal and jejunal lipase activity, suggesting that spermidine can improve intestinal lipid metabolism and has the potential to be developed as a lipase inhibitor. Trypsin is a type of hydrolase that breaks down proteins into peptides (Kaur and Singh, 2022). Our study found that the trypsin content in the duodenum and jejunum remained stable, but spermidine and 3-NPA significantly changed the trypsin content in ileum. It was speculated that the ileum was close to the colon and cecum, and the microbial species increased. Spermidine or 3-NPA affected the trypsin activity by changing the microbial diversity. Compared with lipase and amylase, trypsin was always at a lower level in each intestinal segment. This may be related to the excessive increase in trypsin activity, which may cause a variety of diseases (Li et al., 2014). Spermidine combined with 3-NPA always reduces the activity of digestive enzymes. It is speculated that there may be an interaction between spermidine and 3-NPA, which inhibits endogenous digestive enzyme secretion. The specific mechanism remains to be further studied. In conclusion, feeding spermidine can significantly change the activity of intestinal digestive enzymes, promote the utilization of starch and inhibit the accumulation of fat.

Spermidine removes ROS and is considered an antioxidant (Balderas et al., 2016). Malondialdehyde oxidative stress is the reaction product of lipid peroxidation. MDA content can be used as one of the indices to measure the degree of oxidative stress in the body. Our study found that 3-NPA caused a significant increase in MDA in the intestinal tissues of geese, indicating that 3-NPA also caused oxidative stress in the intestinal tissues. MDA in the ileal tissue of piglets was significantly decreased after 11 days of feed treatment with 0.15% humus (Wu, 2018). This was consistent with our study, which showed that irrigation with spermidine significantly reduced the MDA content in small intestine tissues, and the MDA content in the intestinal tract of the spermidine combined with 3-NPA group was also significantly lower than that in the 3-NPA group. We also found that 3-NPA reduced intestinal spermidine levels, and the addition of spermidine reversed this situation. These results indicated that spermidine could reduce oxidative damage by reducing MDA accumulation. Our study found that spermidine could increase the activities of SOD and GPX enzymes in intestinal tissues, but did not affect the activities of CAT enzymes. The main function of CAT enzymes is to decompose H_2_O_2_ into O_2_ and water (Glorieux and Calderon, 2017). Spermidine has a positive charge at physiological pH and can directly neutralize H_2_O_2_ (Feuerstein et al., 1986). It is speculated that the antioxidant function of spermidine is similar to that of the CAT enzyme, so it does not affect its activity. Our study found that 3-NPA can increase the activities of intestinal SOD and CAT enzymes, while 3-NPA also causes MDA accumulation in intestinal tissues. It is speculated that the activity of antioxidant enzymes can be increased after 3-NPA stimulation, but it is not enough to alleviate the damage caused by 3-NPA. In conclusion, spermidine increased the activities of a variety of antioxidant enzymes and reduced lipid peroxidation, avoiding intestinal tissue damage induced by elevated oxidative stress levels.

Tens of thousands of microbial floras cogrowing in the gut and the host form a complex and changeable microecosystem, playing an important role in host metabolism and immunity (Wang et al., 2014). Changes in microbial flora can enable the host to rapidly adjust its metabolic and immune performance in response to environmental changes (Candela et al., 2012). Exogenous spermidine can increase the abundance of *Fournierella* and *Anaerofilum* flora in the intestinal tract of calves. *Fournierella* is the dominant genus in the intestinal tract of calves, and the abundance of *Fournierella* in the intestinal tract of calves with wet and hot diarrhea decreases. Moreover, *Fournierella* is positively correlated with phosphatidylcholine content (Yan et al., 2022). *Phosphatidylcholine* is one of the important components of intestinal mucus, which is the first barrier against bacterial invasion of the intestine (Stremmel et al., 2012). Qiu et al. showed that *Anaerofilum* in the gut of broilers was positively correlated with the production of short-chain fatty acids (Ali et al., 2022). Other studies showed that adding 1% *Spicarum* powder to the diet could improve the abundance of beneficial bacteria such as *Anaerofilum*, *Sutterella* and *Peptococcus*, improve intestinal morphology and antioxidant capacity, and thus improve intestinal health level and production performance of broilers (Zhang et al., 2022). We speculated that spermidine could affect the intestinal barrier and metabolism and improve intestinal health by increasing the abundance of beneficial bacteria.

Compared with the control group, the abundance of *Oscillospiraceae*, *Parabacteroides* and *NK4A214* decreased significantly after 3-NPA treatment. *Collinsella* and *Faecalitalea* show significant increases in abundance. *Oscillospiraceae* is a genus of anaerobic bacteria belonging to the phylum Firmicutes and the family *Ruminococcaceae*. An increase in vitamin D content decreases the abundance of *Rumenococcaceae* (Bellerba et al., 2021). Other studies have shown that lipids and fatty acids play an essential regulatory role in intestinal absorption of vitamin D (Pyne et al., 2016). Our study found that after treatment with 3-NPA, the abundance of certain species of Spirillum in the gut decreased, while treatment with spermidine had no effect on it. It is hypothesized that 3-NPA therapy reduces the abundance of *Oscillospiraceae* in the intestine, affecting lipid metabolism and other pathways, and thus intestinal vitamin D absorption. *Collinsella* can participate in the regulation of cholesterol absorption, glycogen synthesis and triglyceride metabolism. Chen et al. showed that *Collinsella* could increase the expression of interleukin-17 and other inflammatory factors, causing intestinal inflammation and damaging intestinal barrier function. Other studies have shown a positive correlation between *Collinsella* and serum triglycerides and the degree of cirrhosis. In this study, it was found that 3-NPA treatment significantly increased the abundance of *Collinsella*, and it was speculated that 3-NPA treatment would lead to the disturbance of lipid metabolism in the body, and then cause intestinal inflammation or liver diseases (Taneja, 2016). The abundance of the *gram-negative pathogen Parabacteroides* increases the permeability of the intestinal epithelium and leads to inflammation and endotoxemia. *Parabacteroides* is also involved in carbohydrate and insulin signaling pathways (Hasain et al., 2020). Other studies have shown that *Parabacteroides* can produce acetate, reduce neutrophil infiltration and alleviate acute pancreatitis (Lei et al., 2021). Our study found that 3-NPA significantly decreased the abundance of *Parabacteroides*, suggesting that 3-NPA may affect metabolic pathways and intestinal epithelial cell permeability. *Faecalitalea* belongs to the *Dantobacter* family and is associated with intestinal inflammatory diseases (Dai et al., 2020). Wang et al. (2021) showed that adding xylanase to broiler diets could reduce the abundance of harmful bacteria such as *Faecalitalea* and *Shigella Castellani*, and promote the colonization of beneficial bacteria, thereby improving the growth performance of broilers. After 3-NPA treatment, the abundance of *Faecalitalea* was significantly increased, indicating that 3-NPA may cause intestinal inflammation and affect growth performance. *NK4A214* belongs to the rumen bacteria family, which can decompose plant cellulose and hemicellulose in the gut and produce short-chain fatty acids to provide energy for the host (Wang et al., 2019). *NK4A214* is a potential beneficial intestinal bacterium (Kan et al., 2021). Liang et al. (2021) showed that *NK4A214* was negatively correlated with piglet diarrhea. This study found that spermidine and 3-NPA treatment groups significantly reduced the abundance of *NK4A214*, and the abundance of *NK4A214* in the gut of obese mice also decreased (Zhao et al., 2017). The results showed that spermidine and 3-NPA could inhibit the colonization of *NK4A214* and reduce the production of short-chain fatty acids. Compared with the 3-NPA group, the abundance of *Barnesiella* in the 3-NPA + SPD group significantly increased, and the abundance of *Eubacterium* significantly decreased. With the use of antibiotics, curbing the emergence and spread of resistant bacteria has become a major challenge (Snitkin et al., 2012). Vancomycin-resistant Enterococcus (VRE) is a type of Gram-positive coccus with strong Vancomycin resistance. Ubeda et al. (2013) showed that *Barnesiella* colonization could improve intestinal resistance to VRE colonization, limit its growth, and thus regulate intestinal microbial composition. Other studies have shown that the addition of resistant starch can increase the abundance of *Barnesiella* in the intestines of mice, thereby inhibiting intestinal inflammation (Hu et al., 2016). This study found a significant increase in the abundance of *Barnesiella* in the combined treatment group as compared to the 3-NPA group, suggesting that spermidine may be able to modify the structural changes in the gut flora caused by 3-NPA to some extent and inhibit 3-NPA-induced inflammation in the gut. *Eubacteriu*m is a kind of Gram-positive bacillus with uniform size, which can decompose carbohydrates to produce short-chain fatty acids (Ghosh et al., 2020). As a healthy diet, the Mediterranean diet can increase the abundance of *Eubacterium* (Mediterranean diet intervention alters the gut microbiome in older people reducing frailty and improving health status: the NU-AGE 1-year dietary intervention across five European countries). *Eubacterium* can degrade complex carbohydrates and produce short-chain fatty acids, then regulate intestinal inflammation or colonize in intestinal mucous layer and improve the utilization capacity of intestinal short-chain fatty acids (Van den Abbeele et al., 2013; Chen et al., 2015). Interestingly, the combined treatment group showed a significant reduction in *Eubacterium* abundance compared to the 3-NPA group, suggesting that spermidine may inhibit colonization of some *Eubacterium* genera in the gut. In summary, spermidine modulates the structure of the gut flora and promotes the colonization of beneficial bacteria in the gut. 3-NPA can damage the body by promoting the colonization of harmful bacteria and damaging intestinal health.

Gut microbes are considered to be one of the body organs that can affect gut metabolism and health, and metabolites produced by their decomposition of food are also key substances in regulating the host state. Spermidine promotes the production of beneficial metabolites. *Wikstromol* has anti-inflammatory effects (Yatkin et al., 2014). Laavola et al. showed that *Wikstromol* could relieve the inflammation of the limbs of mice and inhibit the production of inflammatory factors in macrophages (Laavola et al., 2017). The content of *Wikstromol* increased after spermidine treatment, which suggested that spermidine might have an anti-inflammatory effect on the intestinal tract. *Acetoeugenone* (*AS 1–5*) is one of the phenolic compounds produced by the degradation of lignin (Liu et al., 2020). The content of *AS 1-5* in the SPD group was significantly increased, indicating that spermidine was conducive to lignin decomposition and could be used as an additional additive for microbial enzymatic hydrolysis of lignin (Kamimura et al., 2019). *Alpha-bisabolol* is a sesquiterpene alcohol that has anti-inflammatory, anti-apoptotic and antibacterial effects (Ramazani et al., 2022). Spermidine can increase the *Alpha-bisabolol* content in the intestine, suggesting that spermidine can exert anti-inflammatory and anti-apoptotic effects by regulating the *Alpha-bisabolol* content. Dietary supplementation with *Flavine mononucleotide* can improve the anti-inflammatory and antioxidant capacities of the body (Suwannasom et al., 2020). This study found that the content of *Flavine mononucleotide* decreased significantly after spermidine treatment. However, the activity of intestinal glutathione reduction was significantly increased, suggesting that spermidine might enhance the activity of antioxidant enzymes and inhibit the production of *Flavine mononucleotide* in other ways. The specific mechanism of action remains to be further studied.

Spermidine promotes the production of beneficial metabolites that improve gut quality. 3-NPA affects the production of many metabolites and can cause intestinal damage. *Mannose* plays an essential role in the body’s metabolism and glycosylation of specific proteins. *Mannose* ingestion is accumulated in the form of *D-Mannose 6-phosphate* (Gonzalez et al., 2018). Zhang et al. (2017) showed that adding *Mannose* to drinking water of mice could promote the proportion of regulatory T cells and inhibit the occurrence of diabetes and airway inflammation. It was found that 3-NPA resulted in a significant reduction in the amount of *D-Mannose 6-phosphate* in the gut, it is speculated that 3-NPA affects mannose uptake and may cause inflammation in the gut. *Butany-L-carnitine* can be transported to the intestine for absorption by the carnitine transprotein OCTN2, and the impairment of OCTN2 function may lead to intestinal inflammation (Vermeire and Rutgeerts, 2005). Srinivas et al. (2007) showed that *Butyryl-L-carnitine* has the potential to treat intestinal inflammation and can provide both butyrate and carnitine. Treatment with 3-NPA significantly reduces the amount of *Butyrate-L-carnitine* in the gut, and it has been hypothesized that 3-NPA affects OCTN2 function and reduces the absorption of *Butyrate-L-carnitine*, which can cause inflammation in the gut. Isodesmosine is a biomarker of chronic obstructive pulmonary disease and systemic inflammation (MacNee, 2013). 3-NPA can promote inflammation by enhancing the expression of *IL-1β* and *TNF-α* (Abdelfattah et al., 2020). Previous laboratory studies have found that intraperitoneal injection of 3-NPA in Sichuan white geese can cause ovarian damage. It has been speculated that 3-NPA promotes elastin hydrolysis, and thus the production of *Isodesmosine*. Meanwhile, 3-NPA increases systemic inflammatory factor levels after entering the bloodstream, causing tissue damage and inducing disease. Studies have shown that *N-Butylscopolamine* can inhibit intestinal motility in mice (Sasaki et al., 2020). Intestinal peristalsis has an impact on the body’s digestive ability and immune defense. After 3-NPA treatment, the increase of *N-Butylscopolamine* content may lead to the inhibition of intestinal peristalsis, affect intestinal flora and thus lead to inflammation. *Blasticidin S* is able to target translation by binding to the peptidyl transferase center of the large subunit of the ribosome and is able to inhibit the translation extension and termination process in some bacteria (Powers et al., 2021). In the 3-NPA group, the content of *Blasticidin S* increased significantly and the abundance of microorganisms decreased. It was speculated that 3-NPA inhibited the growth and colonization of some bacteria by increasing the content of *Blasticidin S*. Under oxidative stress, spermidine has a limited effect on metabolite content. *Ferulic acid 4-sulfate* is a cinnamic acid diffractant that has anti-inflammatory and antioxidant effects. *Ferulic acid* and *Ferulic sulfate* are negatively charged at high pH, and spermidine is a positively charged aliphatic compound (Kok et al., 2014). It is speculated that the interaction of spermidine with *Ferulic acid* leads to a significant decrease in the *Ferulic acid 4-sulfate* content in the intestinal tract. Pyne et al. (2016) showed that phosphorylation of Sphingosine can produce sphingosine phosphate, which can promote the interstitial transformation of epithelial cells. In this study, it was found that the content of Sphingosine increased after spermidine treatment, and spermidine could improve intestinal morphology, suggesting that spermidine might induce the proliferation and differentiation of epithelial cells by promoting sphingosine metabolism. In summary, spermidine and 3-NPA can change the content of intestinal metabolites, spermidine can increase the content of a variety of beneficial metabolites, and 3-NPA can inhibit the production of a variety of beneficial metabolites and thus cause intestinal inflammation.

## 5 Conclusion

**Figure 10:**
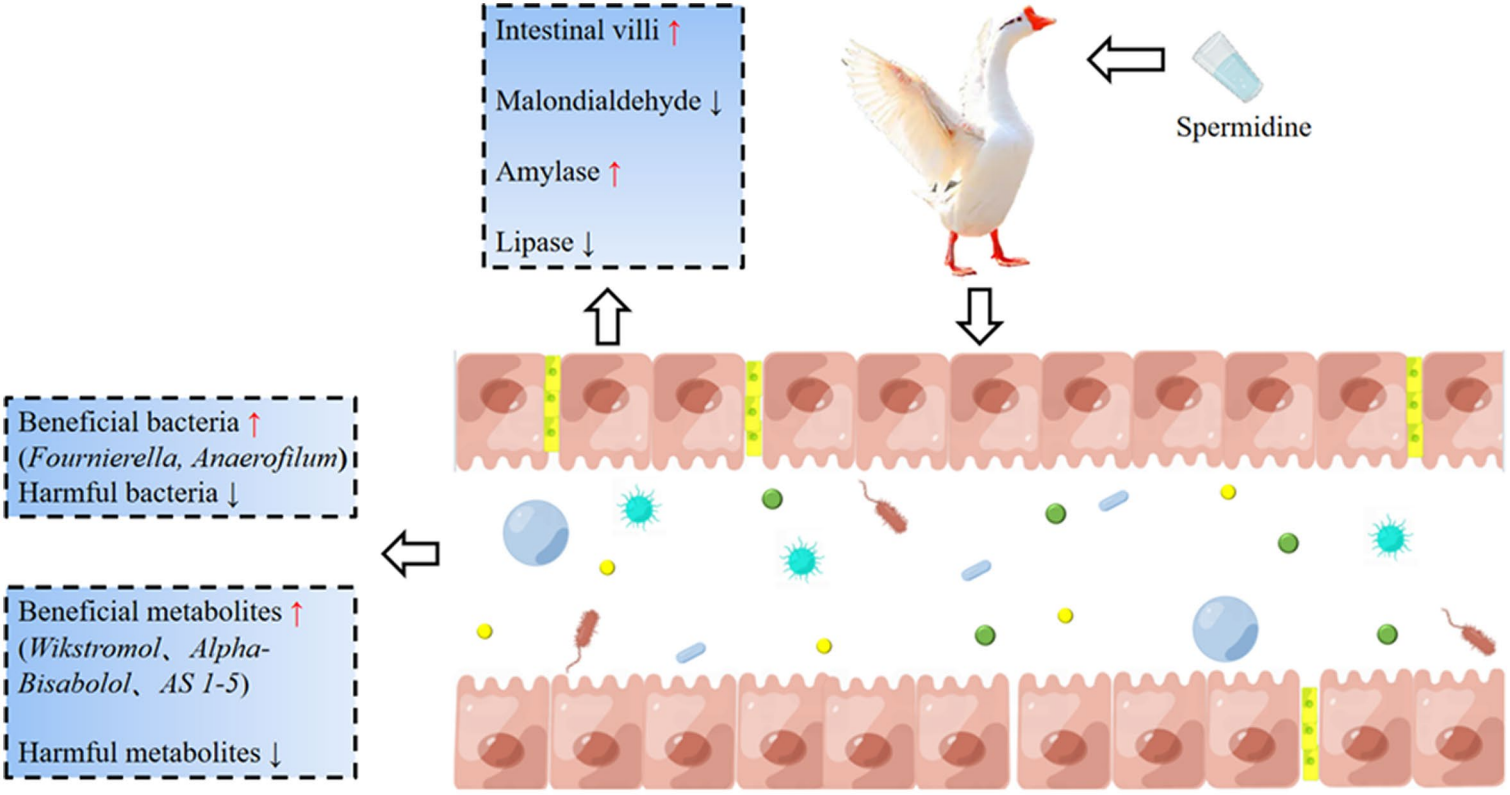
The basic mechanism of spermidine improving intestinal health. Spermidine can improve intestinal morphology, improve SOD, CAT, and GPX enzyme activities, promote the colonization of beneficial bacteria such as *Fournierella* and *Anaerofilum*, and improve intestinal health by producing beneficial metabolites such as *Wikstromol*, *Alpha-bisabolol* and *AS 1-5*. 3-NPA reduces intestinal health and causes intestinal oxidative damage.

Our experiment clarified the effects of spermidine on the intestinal health indices of Sichuan white geese. Spermidine can improve intestinal morphology, improve SOD, CAT and GPX enzyme activities, promote the colonization of beneficial bacteria such as *Fournierella* and *Anaerofilum*, and improve intestinal health by producing beneficial metabolites such as *Wikstromol*, *Alpha-bisabolol* and *AS 1–5*. 3-NPA reduces intestinal health and causes intestinal oxidative damage, which may be related to 3-NPA inhibiting beneficial metabolites such as *D-mannose 6-phosphate* and *Butyryl-L-carnitine* and promoting the production of harmful metabolites such as *Blasticidin S* and *N-butylscopolamine*. In conclusion, spermidine can improve intestinal health and alleviate intestinal damage caused by 3-NPA, which provides basic data for the development of spermidine-rich diets (Figure 10).

## Data availability statement

The original contributions presented in the study are publicly available. This data can be found at: https://www.ebi.ac.uk/metabolights/, MTBLS9084.

## Ethics statement

The animal study was approved by Animal Operation Code and Welfare Committee of Sichuan Agricultural University. The study was conducted in accordance with the local legislation and institutional requirements.

## Author contributions

ZW: Methodology, Writing – original draft. DJ: Funding acquisition, Validation, Writing – original draft. XW: Data curation, Writing – original draft. YJ: Formal analysis, Writing – original draft. QS: Formal analysis, Writing – original draft. WL: Validation, Writing – original draft. XA: Validation, Writing – original draft. CJ: Software, Writing – original draft. SL: Validation, Writing – original draft. YQ: Validation, Writing – original draft. BK: Funding acquisition, Validation, Writing – review & editing.
